# Preparation and characterization of acetylated starch/papain composites

**DOI:** 10.1039/d4ra05814c

**Published:** 2024-11-26

**Authors:** Sasitorn Boonkerd, Hongxun Hao, Lek Wantha

**Affiliations:** a School of Chemical Engineering, Institute of Engineering, Suranaree University of Technology Nakhon Ratchasima 30000 Thailand lekwa@g.sut.ac.th; b National Engineering Research Center of Industrial Crystallization Technology, School of Chemical Engineering and Technology, Tianjin University Tianjin 300072 China

## Abstract

This research aimed to prepare and characterize acetylated starch/papain composites by encapsulating papain within acetylated cassava starch with a low degree of substitution (DS = 0.037) through a stepwise antisolvent precipitation method. The effects of starch concentrations, starch solution volumes, and surfactant types and concentrations were examined. An increase in starch concentration generally enhanced EE, but an excessive concentration led to a decrease in performance due to the aggregation of starch. Furthermore, LC decreased as the starch concentration increased, while the volume of the starch solution primarily influenced LC. Surfactants were employed to disperse the particles and prevent their aggregation during encapsulation, with higher concentrations, particularly of Tween 80, improving both EE and LC but reducing the activity of papain. Optimal results were achieved with a starch concentration of 30 mg mL^−1^, solution volume of 7 mL, and 3% v/v Tween 80, resulting in an EE of 96.23% and LC of 12.40%. However, the residual papain activity under these conditions dropped to approximately 56%. In contrast, Tween 20 at 1% v/v preserved higher papain activity (87%), although it yielded a lower EE of 69.87% and LC of 9.32%. SEM images revealed that the resulting composite particles had rough, indistinct clusters with surfaces featuring clustered starch nanoparticles. Confirmatory analyses *via* fluorescence spectra and FTIR confirmed successful entrapment of papain within acetylated starch with a lower degree of substitution.

## Abstract 2

Acetylated cassava starch/papain composites were prepared by a stepwise antisolvent precipitation method.

## Graphical abstract

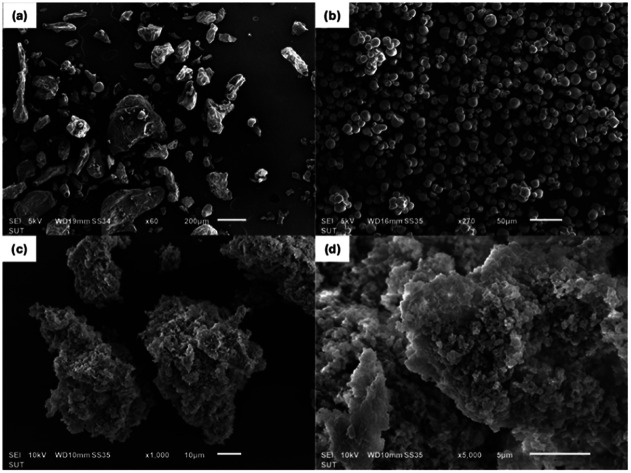

## 1. Introduction

Papain or papaya proteinase I (EC 3.4.22.2), an enzyme that is extracted from papaya (*Carica papaya*), is primarily sourced from the latex of the papaya plant, which can be found in its leaves, stems, and unripe fruits.^1^ This enzyme consists of 212 amino acids.^2^ The versatility of papain extends across various industries, including food processing, cosmetics formulation, detergent manufacturing, leather processing, and pharmaceutical production.^3^ Also, its enzymatic properties make it valuable in numerous applications, such as wound healing,^4^ anti-inflammatory formulations,^5^ antibacterial treatments,^6^ and it even exhibits antioxidant properties.^7^ Furthermore, papain has shown promise in cancer therapy^8^ and as an aid in digestion.^9^ Despite its wide range of benefits, the use of papain in medical or pharmaceutical applications, as well as in food, faces several limitations due to its chemical instability, low bioavailability, and pH instability under acidic conditions (pH below 2.8).^10,11^

Encapsulation is one of the methods to produce composite particles, which is a process of enclosing substances within a carrier material to protect them from external factors, such as heat, moisture, and oxidation, thereby enhancing their stability and prolonging their shelf life.^12^ In the pharmaceutical industry, encapsulation is commonly used to deliver drugs in a controlled manner, ensuring targeted release and improved efficacy while minimizing side effects.^13^ Similarly, in the food industry, encapsulation can preserve flavors, vitamins, and nutrients, preventing their degradation during processing or storage.^12^ Overall, encapsulation offers versatile solutions for various applications, ranging from drug delivery and food preservation to cosmetics and agriculture. Therefore, encapsulation is a method that can help improve the chemical stability and oral bioavailability of papain for various applications.

Carrier materials come in various forms, including synthetic polymers, biopolymers, and inorganic porous materials.^10,14,15^ The choice of carrier materials is essential to the properties of particles. In the encapsulation of papain, various materials have been used to protect and control the release of papain, such as chitosan-reinforced alginate,^16^ poly(ε-caprolactone),^2^ poly(lactic-*co*-glycolic acid) (PLGA),^10^ PVA nanofibers,^17,18^ hydroxypropyl methylcellulose phthalate (HPMCP), Eudragit L 100 and Eudragit S 100.^19^ However, to the best of our knowledge, no publications have been devoted to the encapsulation of papain using starch as a carrier material.

Starch has gained popularity as an encapsulation material for bioactive compounds in the food and biomedical fields due to its natural and renewable nature, biodegradability and biocompatibility.^20,21^ It has been successfully used to encapsulate a range of substances, including curcumin,^22,23^ catechin,^24^ luteolin,^25^ diclofenac sodium,^26^ quercetin,^27^ zeaxanthin,^21^ ciprofloxacin^15^ and bovine serum albumin (BSA).^28^ The ability of starch to protect and deliver these bioactive compounds, while maintaining their stability and functionality has made it an excellent choice for encapsulation. With its numerous benefits, starch has become an increasingly attractive option as an encapsulation material in various applications, while also protecting its encapsulated contents from adverse environmental conditions.

Various types of starch are used for encapsulation, such as native starch,^21,27,28^ OSA starch,^29^ acetylated starch,^15,28^ oxidized starch,^25^ cross-linked starch,^26^ and other types of modified starch.^20^ Among them, native starch offers several advantages, such as environmental friendliness, biocompatibility, and non-toxicity.^20,21^ However, it may not be suitable for controlling drug release due to its tendency to rapidly release drugs.^28,30^ This rapid release is attributed to the high swelling of native starch granules and their susceptibility to enzymatic digestion in biological fluids.^15,28^ Therefore, if controlling drug release is desired, modified starches are of interest given that they offer better control over drug release.^28^

Acetylated starch is one of the modified starches capable of controlling drug release, given that its modification helps reduce its swelling and improves its resistance to enzymatic digestion compared to native starch.^22,28^ However, the properties of acetylated starch depend on the degree of substitution (DS).^15,28^ The higher its DS, the better it inhibits swelling and enzymatic digestion.^28^ However, most factories in Thailand can only produce acetylated starch with a low DS. Thus, to increase its value, this study selected low-DS acetylated starch as the encapsulation material.

The encapsulation of proteins in coating particles can be achieved through various methods, such as emulsion evaporation/extraction, solvent evaporation, interfacial, physical absorption, antisolvent precipitation, and supercritical fluid antisolvent precipitation.^31–33^ In this study, antisolvent precipitation was employed to encapsulate papain with acetylated starch. This method stands out due to its simplicity, quickness, and ease of operation, requiring no extended shear or stirring rates, sonication, or very high temperatures. Additionally, it offers a high encapsulation efficiency with low power consumption.^34^

The encapsulation of papain is very important to improve its stability, and thus the encapsulation process is primarily studied in this research for preparing acetylated starch/papain composites. This work aimed to design and fabricate a papain delivery system by encapsulating papain with acetylated cassava starch (ACS) with a lower DS using a stepwise antisolvent precipitation method and determine the effect of the concentration and volume of the starch solution including the type and concentration of surfactant used for the encapsulation of papain. The chosen surfactants, Tween 20 and Tween 80, are both biocompatible and widely used in the food and pharmaceutical industries.^35^ This selection ensures the safety of the encapsulation process and its potential future applications. Our analysis focused on evaluating the effect on encapsulation efficiency, enzyme loading capacity, and residual activity of papain. The ACS/papain composite was characterized utilizing diverse techniques including fluorescence spectroscopy, Fourier transform infrared spectroscopy (FTIR), and scanning electron microscopy (SEM). These methodologies aim to ascertain the success of papain encapsulation within acetylated cassava starch (ACS) at lower DS, providing insights into the effective coating of papain.

## 2. Materials and methods

### 2.1 Materials

Papain powder (GRM058, *M*_w_ = 23 kDa) and l-cysteine hydrochloride monohydrate (GRM046) were purchased from HiMedia (Nashik, India). Acetylated cassava starch (ACS) with a low DS (DS = 0.037 and acetyl content = 0.96%) was obtained from Sanguan Wongse Industries Co., Ltd, Nakhon Ratchasima, Thailand. Ethanol was purchased from Duksan (Gyeonggi-do, Korea). Tween 20 (T20) and Tween 80 (T80) were purchased from Loba Chemie™ (Mumbai, India). *N*α-Benzoyl-dl-arginine 4-nitroanilide (BAPNA) was purchased from Sigma-Aldrich (Saint-Louis, Switzerland). Dimethyl sulfoxide, sodium hydroxide, and ethylenediaminetetraacetic acid were purchased from RCl-Labscan (Bangkok, Thailand). All other chemicals and reagents used were of analytical grade.

### 2.2 Preparation of acetylated cassava starch (ACS) solution

An ACS solution was prepared using a two-step process, involving heating/gelatinization and subsequent ultrasonication, following a well-established method.^36–38^ Briefly, acetylated cassava starch, dispersed in DI water at a concentration of 100 mg mL^−1^, underwent gelatinization in a shaking water bath at 90 °C for 30 min, with shaking at a rate of 180 rpm. Subsequently, the gelatinized starch paste was cooled to approximately 60 °C. Following this, a 10 min ultrasonication process was performed using a kHz ultrasonic processor (Branson SFX250 Digital Sonifier, Branson Ultrasonics, USA) equipped with a probe transducer featuring a flat tip of 1/2′′ (13 mm). The ultrasonication, conducted at 60% amplitude with a pulse function (5/2 s on/off) to minimize heat generation, was used to ensure the homogeneity of the starch solution. Subsequently, this solution was further diluted to concentrations of 10, 15, 30, and 60 mg mL^−1^ in preparation for the subsequent encapsulation process.

### 2.3 Preparation of ACS/papain composites

The ACS/papain composites were prepared using the stepwise antisolvent precipitation^39^ with minor modifications, and ethanol was used as the antisolvent. Papain was initially precipitated from a 1 mL aqueous solution with a concentration of 30 mg mL^−1^ by adding ethanol solution containing different surfactants (Tween 20 and Tween 80) at various concentrations (1%, 2%, and 3% v/v). This step was carried out at a volume ratio of 1 : 8 (papain solution to ethanol solution). Subsequently, acetylated cassava starch (ACS) solutions at varying concentrations (10, 15, 30, and 60 mg mL^−1^) and volumes (1, 3, and 7 mL) were added to the papain suspension. This mixture was stirred for 30 min at 500 rpm to achieve a homogenous dispersion. Homogenization using a homogenizer at 8000 rpm for 1 min was conducted to facilitate the settling of fine particles in the colloidal suspension in ethanol–water systems. The resulting sediments were collected by centrifugation (BKC-TH16RII, BIOBASE, China) for 10 min at 3000 rpm. They were washed with ethanol for dehydration, and subsequently filtrated using a 0.25 μm membrane using a vacuum pump. After filtration, the ACS/papain composites were dried in a desiccator to remove any remaining moisture and dry the composite.

### 2.4 Preparation of standard curve of papain concentration

The standard curve for papain was created utilizing various concentrations of papain, and subsequently the corresponding absorbance values were recorded at 278 nm. This approach allows the establishment of a reliable relationship between the concentration of papain and its absorbance, providing a basis for accurate quantification in subsequent analyses. This calibration curve was previously established and reported in our previous work.^40^

### 2.5 Encapsulation efficiency (EE) and loading capacity (LC)

The encapsulation efficiency (EE) and loading capacity (LC) of the ACS/papain composites were determined by measuring the absorbance of free papain in the collected solution at 278 nm using a spectrophotometer (DR6000, Hach, USA). Concurrently, a blank sample, comprised of composites without papain but with an identical composition to the test sample, was subjected to the same procedure as the test sample, mitigating potential contributions from other components to the absorbance. Finally, the papain content was quantified against the standard calibration curve of papain in DI water. The EE and LC of papain were computed using the following equations:^2,23^1
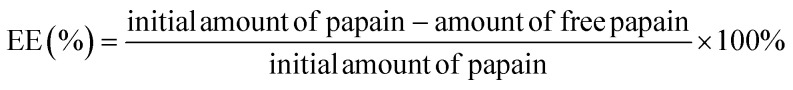
2
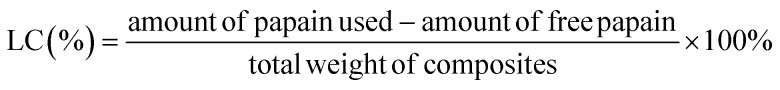


The method for determining the amount of free papain (unencapsulated papain) varied depending on the volume of starch solution used in the encapsulation process. For starch solution volumes of 1 and 3 mL, the free papain was quantified by combining the free papain in the supernatant with the free papain obtained by washing the dried precipitate twice with an ethanol solution (1 : 1 water : ethanol with surfactant). Conversely, for a starch solution volume of 7 mL, only the supernatant obtained after centrifugation was used to determine the amount of free papain.

### 2.6 Enzyme activity measurement

For the evaluation of enzyme activity based on Arnon Ruth's methodology (1970),^17,41^ 1 mL of free-papain solution (1 mg mL^−1^) or papain sample solution was placed into test tubes. Then, 5 mL of precisely measured substrate solution (43.5 mg of BAPNA in 1 mL of dimethyl-sulfoxide and the volume was adjusted to 100 mL with 0.05 M Tris buffer, pH 7.5 containing 0.005 M cysteine and 0.002 M EDTA) was added. Following a 25 min incubation at 298.15 K, the enzymatic reaction was terminated by adding 1 mL of 30% acetic acid. Then, quantification of the liberated *p*-nitroaniline was conducted *via* spectrophotometric analysis at 410 nm (SP-UV 200, Spectrum Instruments, China). Notably, the control tubes without enzyme demonstrated the absence of self-hydrolysis.

Units and specific activity: The enzymatic activity of papain was quantified based on substrate hydrolysis. BAPNA activity is defined as the enzyme hydrolyzing 1 micromole of substrate per minute (*E* = 8800). This is calculated using the equation described by I. E. Moreno-Cortez *et al.*, as follows:^17^3
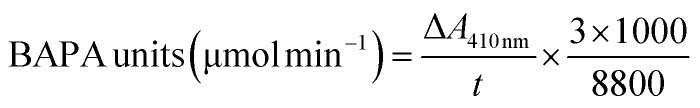
where Δ*A*_410nm_ is the absorbance at 410 nm, *t* is the time in min, which is the duration of the enzymatic reaction, and 8800 M^−1^ cm^−1^ is the *p*-nitroanilide molar extinction coefficient at 410 nm. Specific activity is expressed as units per milligram of protein.

The residual activity (RA) was determined by comparing the activity of the enzyme after the process to its initial activity. This was calculated using the following equation:^42,43^4
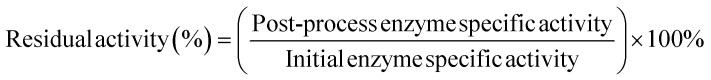


The specific activity of the enzyme was first measured before the process, and post-process activity was determined under the same conditions. The residual activity is reported to reflect the retention of the enzyme function after the process.

### 2.7 Particle characterization

Papain and the papain-loaded starch particles were analyzed using a fluorescence spectrometer (FP-8300, JASCO Corp., Japan). The fluorescence spectra of pure papain (1 mg papain dissolved in 1 mL of DI water, and then mixed with 8 mL ethanol) and ACS/papain composite dispersions were recorded. Excitation was performed at 280 nm, and the emission spectra were obtained between 290 nm and 450 nm using a quartz cell with a path length of 10 mm. Both the excitation and emission bandwidths were set at 5 nm. All data were collected at room temperature.

The morphology of the particles was examined using scanning electron microscopy (SEM) and field emission scanning electron microscopy (FE-SEM). The SEM and FE-SEM analyses were performed using JEOL JSM-6010LV and JEOL JSM-7800F models, respectively (JEOL Ltd, Tokyo, Japan). The observations were conducted at an accelerating voltage of 10 kV and 3 kV for SEM and FE-SEM, respectively. During the analysis, the surface of the samples was sputter-coated with a gold layer for SEM and a carbon layer for FE-SEM to avoid charging.

The Fourier transform infrared spectroscopy (FTIR) was employed to analyze the functional groups on the surface of particles. The Fourier transform infrared (FTIR) spectra for papain, ACS, ACS composites and ACS/papain composites were recorded using a Fourier transform spectrophotometer (Tensor 27, Bruker, Germany) in the range of 4000 to 400 cm^−1^ at a resolution of 2 cm^−1^. To obtain the baseline adjustment.

### 2.8 Statistical analysis

All the experimental results were conducted in triplicate. The results are expressed as mean ± standard deviation (*n* = 3) and analyzed by one-way ANOVA with Tukey's multiple comparison test. The significance was set at *p* < 0.05.

## 3. Results and discussion

In previous studies, Morales-Cruz *et al.*^44^ adapted the original one-step antisolvent precipitation method reported by Fessi *et al.*^45^ into a two-step antisolvent precipitation method for encapsulating proteins with limited solubility in organic solvents within polymer particles. They encapsulated proteins such as lysozyme and α-chymotrypsin within poly(lactic-*co*-glycolic) acid (PLGA) nanoparticles, where the protein was first precipitated to ensure its stability post-encapsulation. Similarly, Nelemans *et al.*^46^ applied the same method with bovine serum albumin (BSA). In their approach, the protein was precipitated with acetonitrile (ACN), followed by the addition of PLGA in ACN solution into the protein suspension. Then, PLGA was precipitated using water containing a surfactant to aid in its dispersion, which served as an antisolvent for PLGA. In this study, a similar approach was employed to precipitate papain to maintain its stability during encapsulation. However, the current study focused on acetylated cassava starch with a low degree of substitution (DS = 0.037), which does not dissolve in organic solvents such as PLGA. Consequently, the encapsulation process was modified using the stepwise antisolvent method reported by Yang *et al.*,^39^ involving the preparation of a starch solution in water (the same solvent used for papain), heating and gelatinizing the starch, and then ultrasonically reducing its viscosity. Therefore, the encapsulation process in this study consisted of two steps, where initially papain was precipitated, followed by the addition of the starch solution to encapsulate the papain nanoparticles formed in the first step.

### 3.1 Papain precipitation

In the first step of papain encapsulation by stepwise antisolvent precipitation, the effects of various parameters on papain precipitation were investigated, as detailed in our previous report.^40^ The effects of different parameters such as the type of antisolvent, solvent-to-antisolvent volume ratios, and the papain concentration were examined. The primary role of the antisolvent is to reduce the solubility of papain in the solution, leading to its rapid precipitation. The antisolvent induces a high degree of supersaturation, which increases the nucleation rate. This increased nucleation rate leads to the continuous development of small, amorphous particles.^47,48^ The choice of solvent and antisolvent is very important. Water was used as the solvent because papain has good solubility in water. Regarding the antisolvent, three types of organic solvents were used, acetone, acetonitrile, and ethanol, given that papain is sparingly soluble or insoluble in these solvents. Additionally, the solvent and antisolvent must be miscible to ensure the effective precipitation of papain.^1,40,48^

**Fig. 1:**
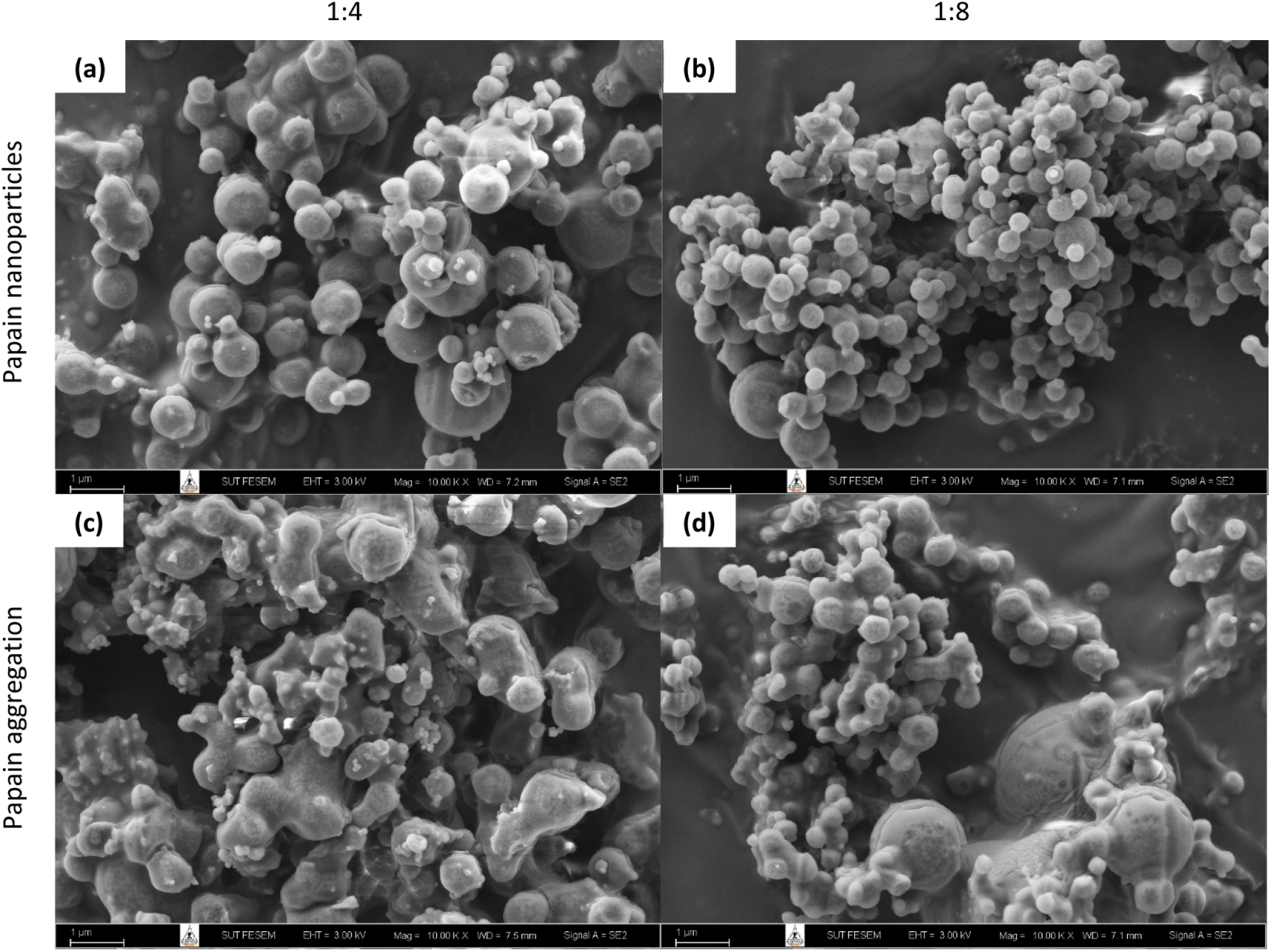
SEM photomicrographs of papain precipitated at different solvent-to-antisolvent ratios: papain nanoparticles (a) 1 : 4 and (b) 1 : 8, and papain aggregates (c) 1 : 4 and (d) 1 : 8.^40^

The results revealed that ethanol was the most suitable antisolvent for the precipitation of papain because it maintained the highest enzyme activity among the organic solvents. Additionally, ethanol offers a safer and more environmentally friendly option, effectively reducing the solubility of papain in water and leading to rapid precipitation with minimal aggregation. The optimal conditions for the precipitation of papain were found to be at a papain concentration of 30 mg mL^−1^ and a solvent-to-antisolvent volume ratios of 1 : 4 (Fig. 1(a)), where papain maintained its stability at a zeta potential of 35.1 ± 3.6 mV and 100% activity with a particle size of 207.6 ± 2.1 nm.

However, upon further utilization for encapsulation purposes, it was observed that at a 1 : 4 ratio, the papain precipitates tended to aggregate (Fig. 1(c)). Increasing the ratio to 1 : 8 (Fig. 1(b)) resulted in smaller (179.1 ± 1.4 nm) and more uniform papain precipitates with reduced aggregation (Fig. 1(d)) and a relatively high zeta potential (43.9 ± 1.9 mV), suggesting that the precipitates were stable and suitable for encapsulation with starch. Despite this, a higher ratio (1 : 8) at 30 mg mL^−1^ of papain also led to the formation of larger particles (more than 1000 nm). Consequently, for the encapsulation experiments, we opted for a papain solution concentration of 30 mg mL^−1^ and a solvent-to-antisolvent volume ratio of 1 : 8 for the initial precipitation step.

### 3.2 Preparation of ACS/papain composites

After identifying the optimal conditions for the precipitation of papain, papain encapsulation was performed to form the ACS/papain composites. In this section, the effects of the concentration and volume of starch on the process were investigated. Moreover, two surfactants, Tween 20 and Tween 80, at different concentrations were tested.

#### 3.2.1 Effect of starch concentrations

The study of the effects of the concentration of starch is crucial in the encapsulation processes. This study investigated starch concentrations in the range of 10 mg mL^−1^ to 60 mg mL^−1^, using Tween 20 as the surfactant at a concentration of 3% v/v and a starch solution volume of 1 mL. Concentrations beyond this range result in the rapid aggregation of the starch particles, making the effective encapsulation of papain difficult.

**Fig. 2:**
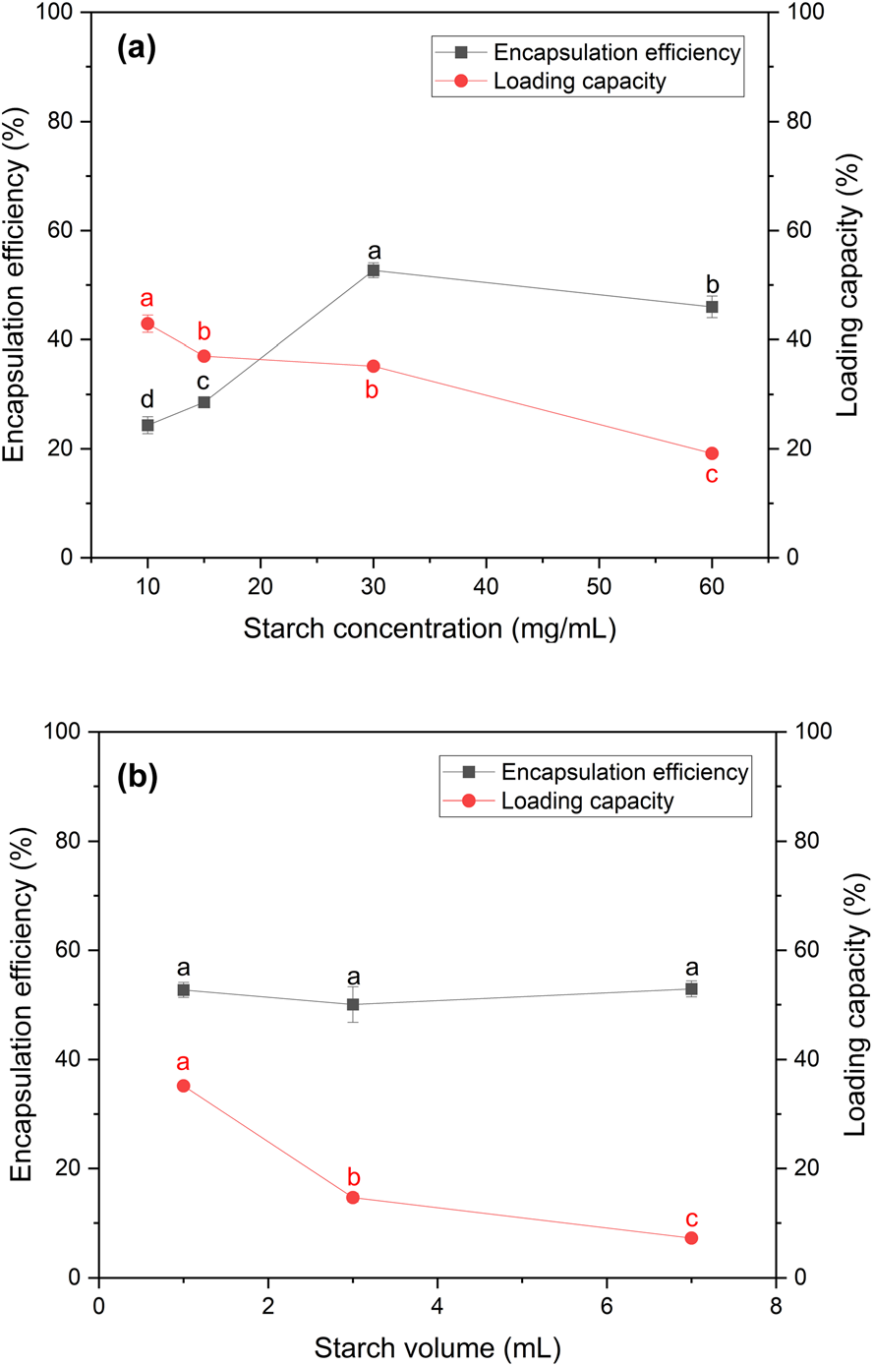
Encapsulation efficiency and loading capacity of ACS/papain composites as a function of (a) starch concentration with fixed volume of starch solution of 1 mL and Tween 20 concentration of 3% v/v, and (b) starch volume with fixed starch concentration of 30 mg mL^−1^ and Tween 20 concentration of 3% v/v. Labels a, b, and c indicate the results of the mean separation method (Tukey's HSD test), which reveals statistically significant differences between the groups.

The results, depicted in Fig. 2(a) illustrate the encapsulation efficiency and loading capacity of the ACS/papain composites as a function of starch concentration. In the range of 10 to 30 mg mL^−1^, the encapsulation efficiency increased significantly from 24.33% ± 1.56% to 52.70% ± 1.38%. However, at 60 mg mL^−1^, the encapsulation efficiency decreased to 45.99% ± 1.97%. This trend is consistent with similar observations for the encapsulation of *Lactobacillus acidophilus* in porous starch.^49^ The decline in encapsulation efficiency at higher starch concentrations is attributed to the increased viscosity of the starch solution at 60 mg mL^−1^, which caused rapid precipitation and aggregation of the starch particles. This higher viscosity inhibited the diffusion between the starch solution and ethanol, resulting in non-uniform molecular supersaturation and slower nucleation rates. Consequently, larger and more aggregated particles were formed,^37,48^ reducing the encapsulation efficiency. Even with the addition of a surfactant to aid their dispersion, these challenges persisted due to the rapid self-aggregation of the starch particles before the effective encapsulation of papain could occur.

Meanwhile, the loading capacity (LC) continuously decreased from 42.89% ± 1.58% at 10 mg mL^−1^ to 19.15% ± 0.67% at 60 mg mL^−1^. The LC at 15 mg mL^−1^ and 30 mg mL^−1^ was quite similar, with values of 37.02 ± 0.60% and 35.18 ± 0.60%, respectively. This continuous reduction is likely due to the increase in the amount of starch as the starch concentration increased, which lowered the amount of papain encapsulated per unit of starch. At higher concentrations, the starch particles tended to aggregate, leading to less effective encapsulation. This aggregation reduced the available surface area for encapsulating papain, and as a result the LC greatly decreased from 35.18% ± 0.60% at 30 mg mL^−1^ to 19.15% ± 0.67% at 60 mg mL^−1^.

Therefore, a starch concentration of 30 mg mL^−1^ was selected for subsequent experiments, given that it provided the highest encapsulation efficiency and a relatively high loading capacity. Although these methods proved to be effective to realize encapsulation, the efficiency of encapsulation decreased with higher starch concentrations. Hence, selecting an appropriate starch concentration is crucial in producing composites with the highest encapsulation efficiency towards papain particles.

#### 3.2.2 Effect of starch solution volumes

To determine the suitable encapsulation efficiency of papain without increasing the starch concentration beyond 30 mg mL^−1^, the volume of starch solution was adjusted. Volumes of 1, 3, and 7 mL were examined, maintaining a starch concentration of 30 mg mL^−1^ and using Tween 20 at 3% v/v as the surfactant. The results shown in Fig. 2(b) demonstrate the encapsulation efficiency and loading capacity of the ACS/papain composites as a function of starch solution volume. The encapsulation efficiency at volumes of 1, 3, and 7 mL was 52.70% ± 1.38%, 50.02% ± 3.28%, and 52.89% ± 1.47%, respectively. These values do not significantly differ from each other, indicating that encapsulation efficiency is relatively stable across different starch volumes. This result suggests that the amount of starch used in these volumes is sufficient to encapsulate the available papain, achieving a saturation point where additional starch does not further enhance the encapsulation efficiency.

However, the loading capacity gradually decreased as the volume of starch solution increased. This reduction occurred because although more starch was used for encapsulation, the amount of papain encapsulated remained the same. A starch volume of 7 mL was chosen as the optimal choice, given that it allows any unencapsulated papain to dissolve back into the solution, making it easier to separate from the final product. This simplifies the purification process, given that dissolved papain can be easily removed by centrifugation or filtration. In contrast, at volumes of 1 and 3 mL, residual free papain remained in solid form, requiring additional steps for its removal, increasing the process complexity and waste. These extra washing steps also raise the risk of papain degradation.

Hence, a starch solution volume of 7 mL seemed the most suitable for subsequent experiments, given that it balances the encapsulation efficiency and loading capacity, while simplifying the purification process and minimizing the risk of papain degradation.

#### 3.2.3 Effect of surfactants

**Fig. 3:**
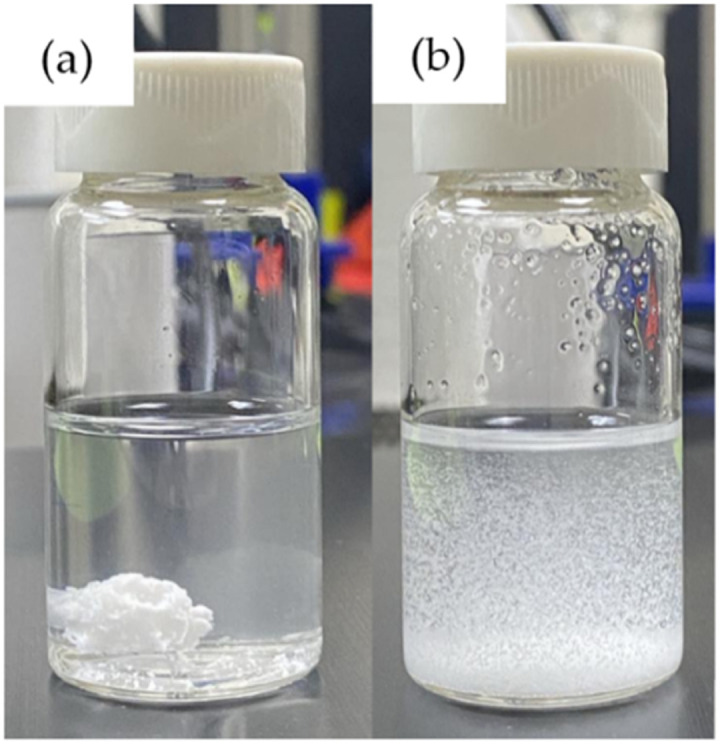
Effect of surfactants on starch particle dispersion: (a) without surfactant and (b) with surfactant.

**Fig. 4:**
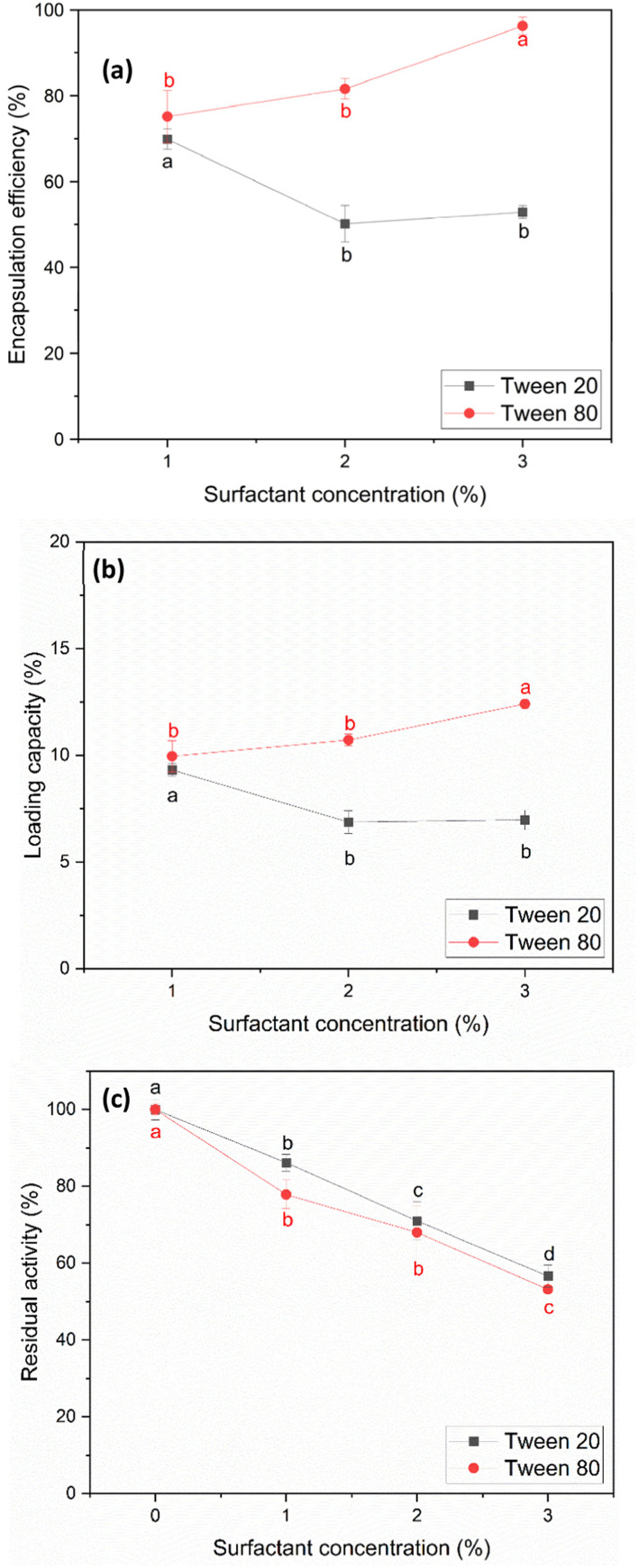
Encapsulation efficiency (a) and loading capacity (b) with fixed starch concentration of 30 mg mL^−1^ and starch solution volume of 7 mL, (c) residual activity after papain precipitation as a function of surfactant concentration (% v/v) and surfactant type. Labels a, b, c, and d indicate the results of the mean separation method (Tukey's HSD test), which reveal the statistically significant differences between the groups.

In the process of encapsulating papain with starch, surfactants such as Tween 20 and Tween 80 were employed to aid in dispersing the starch particles effectively, preventing them from aggregating and forming clumps (Fig. 3).^50^ Besides facilitating particle dispersion, Tween surfactants are commonly studied in pharmaceutical applications to ensure protein stability, as demonstrated in the research by Duskey *et al.*,^51^ who investigated the influence of different types of Tween, 20, 60, and 80, on enzyme activity and stability during the encapsulation process of β-glucosidase (β-Glu) in PLGA nanoparticles. Furthermore, other studies have shown that Tween surfactants can enhance enzyme activity^52^ and protect protein surfaces from denaturation.^53^ However, it is worth noting that although Tween surfactants can enhance the activity of some enzymes, they may also cause a decrease in activity for others, as observed in the studies by Battestin & Macedo^54^ and Kar *et al.*,^55^ which investigated the impact of four types of Tween, Tween 80, Tween 60, Tween 40, and Tween 20, ranging from 0.025–1% v/v on tannase activity. The results indicated that Tween caused a reduction in tannase activity. As shown in Fig. 4(c), both Tween 20 and Tween 80 caused a decrease in papain activity with an increase in their concentration. This reduction in activity is likely due to the interaction between the surfactants and the papain molecule. The fatty acid tails of the Tween molecules, oleic acid in Tween 80 and lauric acid in Tween 20, can bind to hydrophobic regions on the papain surface, potentially disrupting its conformation and hindering its enzymatic activity.^54^ This effect is consistent with the findings observed with tannase in previous studies.^54,55^ However, the system with Tween 20 retained slightly higher activity than Tween 80. This difference can be attributed to the varying lengths and saturation levels of the fatty acid tails, with the shorter, more saturated lauric acid in Tween 20 causing less disruption in the papain molecule than the longer oleic acid in Tween 80.^56^

For studying the effect of Tween 20 and Tween 80 on the encapsulation efficiency and loading capacity, the concentration of both Tween 20 and Tween 80 was limited to 3% v/v. This restriction was implemented because investigations revealed that the activity of papain decreased significantly to 50–60% when the surfactant concentration reached 3% v/v after the precipitation process (Fig. 4(c)). The results in Fig. 4(a) and (b) indicate that both the encapsulation efficiency and loading capacity were influenced by the type and concentration of the surfactants. With Tween 20, the encapsulation efficiency and loading capacity decreased initially within the range of 1% to 2% v/v. This decrease is likely due to the Tween 20 molecules interacting with both papain and starch particles, hindering the encapsulation of papain. At concentrations in the range of 2–3% v/v, the encapsulation efficiency and loading capacity remained relatively constant. This result for Tween 20 is consistent with the findings reported by Duskey *et al.*,^51^ where an increase in the concentration of Tween 20 initially resulted in a decrease in both the encapsulation efficiency and loading capacity, and then they were relatively constant.

Conversely, in the case of Tween 80, both parameters increased as its concentration increased from 1% to 3% v/v. Thus, Tween 80 appears to be more effective at dispersing starch particles and promoting the encapsulation of papain within the particles, which is similar to the findings obtained by Shafie & Fayek *et al.*^57^ This could be due to the longer oleic acid tail in Tween 80, allowing better interactions with both starch and papain.

In the case of residual activity after the encapsulation process, using the result at 3% v/v Tween 80 as an example, the post-encapsulation papain activity remained at 55.70% ± 7.18%. Remarkably, no additional loss in activity was observed after encapsulation compared to the initial precipitation step, where the activity was 53.21% ± 0.94%, indicating no additional activity loss during the final step.

At the highest encapsulation efficiency and loading capacity, Tween 20 achieved 69.87% ± 2.36% and 8.96% ± 0.30%, respectively, at 1% v/v. In comparison, Tween 80 achieved 96.23% ± 2.06% and 12.40% ± 0.23%, respectively, at 3% v/v.

Interestingly, the highest residual activity for Tween 20 occurred at the same point as its peak EE and LC, yielding approximately 87%. In contrast, the highest residual activity of Tween 80 occurred at a concentration of 1% v/v, where it reached approximately 80%, alongside EE and LC values of 75.14% ± 6.10% and 9.95% ± 0.73%, respectively.

### 3.3 Particle characterization

#### 3.3.1 Fluorescent spectroscopy

**Fig. 5:**
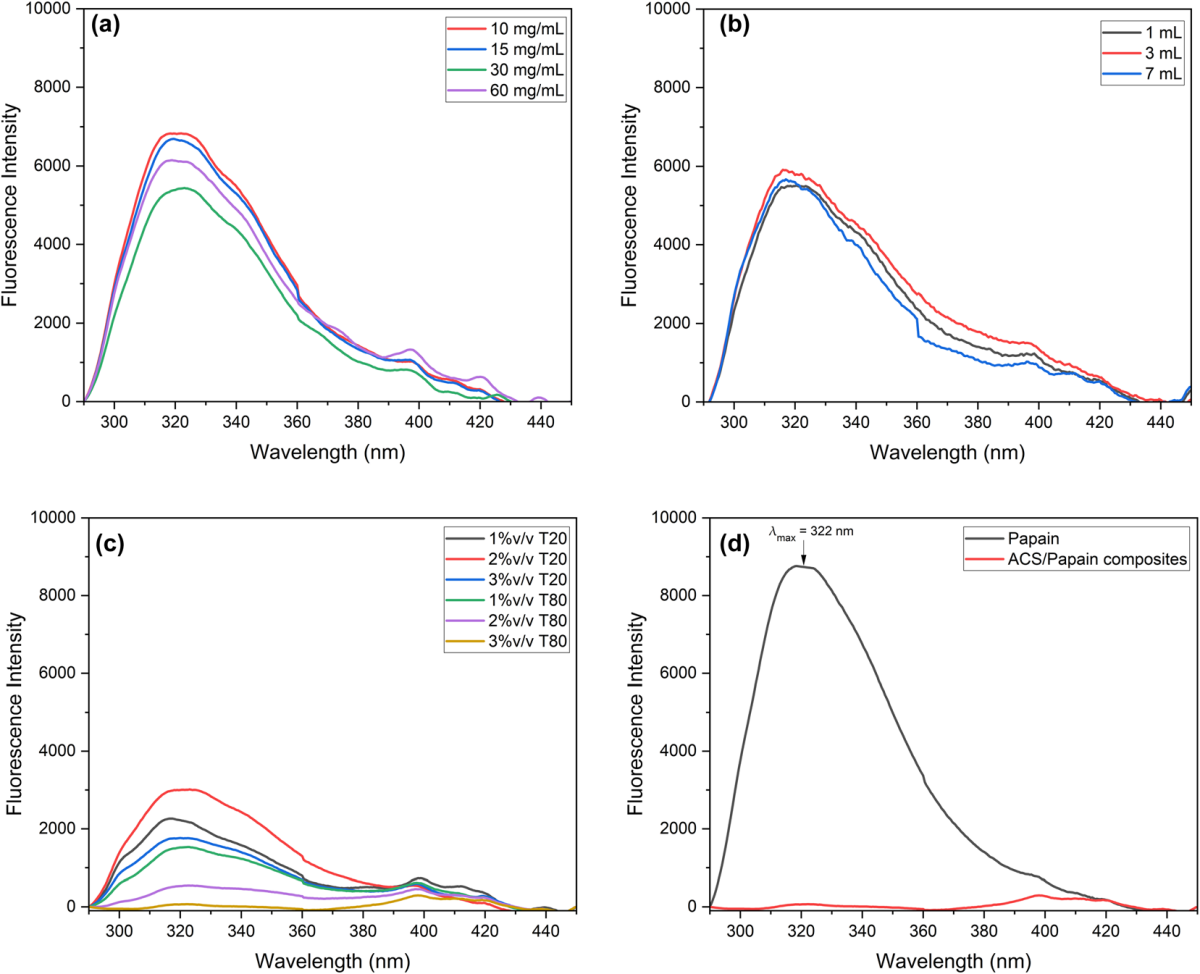
Fluorescence spectra of ACS/papain composites: (a) at different starch concentrations, (b) at different starch volumes, (c) at different surfactant types and concentrations, and (d) under the optimum condition (30 mg mL^−1^, 7 mL, and 3% v/v Tween 80) and pure papain.

Fluorescence spectroscopy analysis was utilized to confirm the encapsulation process. Fig. 5 illustrates the emission spectra of pure papain and ACS/papain composite dispersions upon excitation at 280 nm. In Fig. 5(d), the maximum emission wavelength of papain is located as 322 nm (black spectrum), which is consistent with a previous report documenting it at 332 nm.^2^ The fluorescence intensities of the ACS/papain composites at varying starch concentrations (1 mL) exhibit distinct values, correlating with the encapsulation efficiency (% EE) (Fig. 5(a)). Specifically, as the % EE increased, indicating a higher degree of successful encapsulation of papain within the carrier material, the intensity tended to decrease. This decrease is attributed to the reduction in the presence of free papain molecules, unencapsulated papain molecules, which typically contribute to the overall intensity observed in the solution or solid form. This relationship suggests that ACS encapsulation effectively shielded papain, reducing its availability to emit fluorescence in response to excitation. Conversely, lower % EE values correspond to higher fluorescence intensities, reflecting a greater concentration of free papain molecules.

For differing starch volumes (Fig. 5(b)), the intensity values at the maximum wavelength were similar across the conditions, consistent with their respective % EE values, suggesting that a change in the starch volume does not significantly impact the fluorescence emission, possibly due to the uniform encapsulation efficiency across varying starch volumes. Regarding the surfactant (Fig. 5(c)), the fluorescence intensities at different concentrations of Tween 80 were consistent with the obtained %EE values. However, Tween 20 at 1% v/v, which had the highest %EE, showed a higher intensity compared to Tween 20 at 3% v/v, with a lower % EE. This higher intensity is due to the greater presence of papain on the composite surface at 1% v/v. Conversely, Tween 80 at a concentration of 3% v/v, with the highest % EE of 97.31% ± 1.24%, did not show a peak at the maximum wavelength. Fig. 5(d) indicates that at this highest % EE, no emission maximum was detected (red spectrum), signifying that the papain molecules were effectively encapsulated by ACS with minimal free papain remaining. This supports the successful papain encapsulation within ACS, consistent with the findings of Budama-Kilinc *et al.*^2^

#### 3.3.2 FTIR analysis

**Fig. 6:**
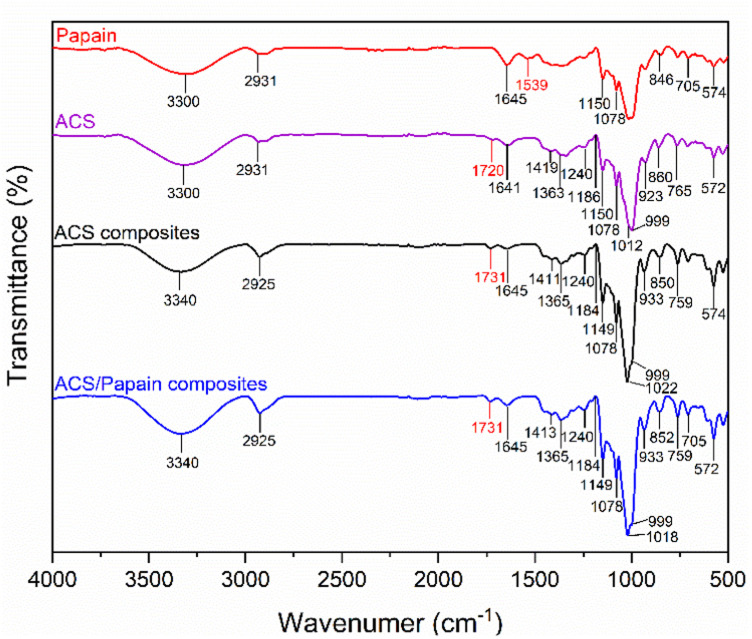
FTIR spectra of papain, ACS, ACS composites and ACS/papain composites.

The FTIR spectra of ACS, papain, ACS composites, and ACS/papain composites are summarized in Table 1, while Fig. 6 illustrate their spectra for comparative visualization.

**Table 1:** FTIR spectral results for papain, ACS and ACS/papain composite

Assignment for papain	References	This study		Assignment for ACS	References	This study		
		Papain	ACS/Pap. com.			ACS	ACS com.	ACS/Pap. com.
N–H stretching of secondary amide bond	3300 (ref. 58)	3300	3340	O–H stretching	3405 (ref. 22)	3300	3340	3340
	3289 (ref. 2)				3388 (ref. 28)			
	3450–3225 (ref. 59)				3421 (ref. 15 and 60)			
					3404 (ref. 21)			
					3000–3700 (ref. 61)			
–CH-asymmetric stretching	2924 (ref. 58)	2931	2925	C–H stretching	2930 (ref. 22)	2931	2925	2925
	2931 (ref. 2)				2930 (ref. 15)			
					2935 (ref. 61)			
–CONH amide I	1637 (ref. 58)	1645	1645	Water adsorption δ(OH) bending	1644 (ref. 21)	1641	1645	1645
	1652 (ref. 2)				1655 (ref. 61)			
	1645.2 (ref. 59)							
–CONH amide II	1551 (ref. 58)	1539	—	Carbonyl C=O stretching vibration	1731 (ref. 22)	1720	1731	1731
	1557 (ref. 2)				1754 (ref. 28)			
					1750 (ref. 15)			
					1760 (ref. 61)			
					1754 (ref. 60)			
CS stretching (sulphide)	1150 (ref. 58)	1150	1149	Carbonyl C–O stretching vibration	1240 (ref. 60 and 61)	1240	1240	1240
	1173 (ref. 2)	1078	1078		1193 (ref. 28)			
	1150–1050 (ref. 18)							
	1076 (ref. 58)							
	1080 (ref. 2)							
				CH_3_ antisymmetric bending vibration	1435 (ref. 15 and 60)	1419	1411	1413
					1373 (ref. 28)			
–CS stretching (disulphide)	852 (ref. 58)	846	852	CH_3_ symmetrical deformation vibration	1245 (ref. 28)	1363	1365	1365
	705–570 (ref. 18)	705–574	705–572		1375 (ref. 15 and 60)			
					1380 (ref. 61)			
				Anhydroglucose ring stretching				
Aromatic residue of tryptophan or tyrosine	868, 850 (ref. 18 and 19)	846	852	C–O bond stretch stretching	1160, 1082, 1017 (ref. 22)	1186, 1150, 1078, 1012	1184, 1149, 1078, 1022	1184, 1149, 1078, 1018
					1193 (ref. 28)			
					1024 (ref. 15)			
					1155, 1086, 1022 (ref. 61)			
					1159, 1082, 1014 (ref. 60)			
				Anhydroglucose ring stretching vibration	992, 930, 862, 763, 574 (ref. 15)	999, 923, 860, 765, 572	999, 933, 850, 759, 574	999, 933, 852, 759, 572
					935, 855, 767, 577 (ref. 61)			
					992, 929, 861, 765, 575 (ref. 60)			

The FTIR spectra of ACS reveal characteristic absorption bands associated with acetylated starch. The peaks at 1150, 1078, and 1012 cm^−1^ correspond to the C–O bond stretching, while the additional characteristic absorption bands at 999, 923, 860, 765, and 572 cm^−1^ are attributed to the stretching vibrations of the anhydroglucose ring, reflecting the basic structural components of starch. The peak at 1641 cm^−1^ is assigned to water adsorption, indicating the presence of bound water within the starch. The band at 2931 cm^−1^ corresponds to the C–H stretching vibrations, and the broad band around 3300 cm^−1^ corresponds to the O–H stretching vibrations, representing both free and inter/intramolecularly bound hydroxyl groups, which play crucial role in the structure of starch. Furthermore, given that the starch used was acetylated starch, additional peaks were observed at 1720, 1419, and 1363 cm^−1^, which are attributed to carbonyl C=O, CH_3_ antisymmetric bending vibration, and CH_3_ symmetry bending vibration, respectively. These additional peaks confirm the introduction of acetyl groups into the starch granules.

The FTIR spectrum of papain shows a broad absorption band at 3300 cm^−1^, corresponding to the N–H stretching of the secondary amide bonds, representing the protein backbone. The peak at 2931 cm^−1^ is associated with the –CH_2_– asymmetric stretching. The amide-I and amide-II bands were observed at 1645 cm^−1^ and 1539 cm^−1^, respectively, confirming the protein secondary structure, as reflected in the peptide bond vibrations. Papain also showed characteristic peaks between 1150, 1078, 846 cm^−1^ and 705–574 cm^−1^, which are attributed to the sulphide and disulphide (–CS) stretching vibrations. Additionally, the peak observed at 846 cm^−1^ is also attributed to the aromatic residues of tryptophan or tyrosine.

In contrast, the FTIR spectra of the ACS composites that were subjected to the process without papain loading but with the inclusion of Tween 80 showed peaks closely aligned with ACS, the raw material. However, noticeable changes in their peak intensity and position were observed. Specifically, the carbonyl C=O peak became sharper and shifted from 1720 to 1731 cm^−1^, and the C–O bond stretching peak shifted from 1012 to 1022 cm^−1^ and became more pronounced. These changes likely result from the addition of Tween 80 and subsequent reprecipitation, which could induce alterations in the structure and properties of ACS. The O–H stretching vibration shifted from 3300 to 3340 cm^−1^, indicating stronger hydrogen bonding within the matrix, which is possibly due to the interactions with the surfactant and cross-linking.

Upon comparing the spectra of papain, ACS composites, and ACS/papain composites, it was observed that many papain peaks overlapped with that of the ACS composites. However, the distinguishing peaks between the ACS composites and papain are the amide-II peak (1537 cm^−1^) for papain and the carbonyl C=O peak (1731 cm^−1^) for the ACS composites. In the spectra of the ACS/papain composites, the amide-II peak of papain is absent, while the carbonyl C=O peak for the ACS composites is present. This absence of the amide-II peak suggests that papain was encapsulated within the ACS without remaining in its free form. Previous studies reported similar results,^2,22,24,25^ where the peak representing the core material disappeared or decreased in intensity, confirming the successful encapsulation. Additionally, the increased intensity of the amide-I peak (1645 cm^−1^) and the O–H stretching (3340 cm^−1^) compared to the ACS composites indicates interactions between the papain and ACS, likely through hydrogen bonding between the hydroxyl (–OH) and carboxyl groups (–COOH) of ACS and the amide groups of papain. This further confirms the enhanced hydrogen bonding in the ACS/papain composites, supporting the successful encapsulation of the enzyme.

Other studies have used starch as a coating material and reported similar encapsulation behavior for the active ingredients and starch, where the hydrogen bonding between the hydroxyl and carboxyl groups facilitated encapsulation.^15,22,62^ In this study, the encapsulation of papain in ACS was achieved through similar interactions. The hydroxyl (–OH) and carboxyl (–COOH) groups on ACS form hydrogen bonds with the functional groups on papain, such as the amide groups, facilitating the entrapment of papain within the ACS matrix. These hydrogen bonds not only aid in the encapsulation process but also contribute to maintaining the structural integrity and activity of the enzyme, enhancing the overall efficacy of the encapsulation.

#### 3.3.3 Morphology of the particles

**Fig. 7:**
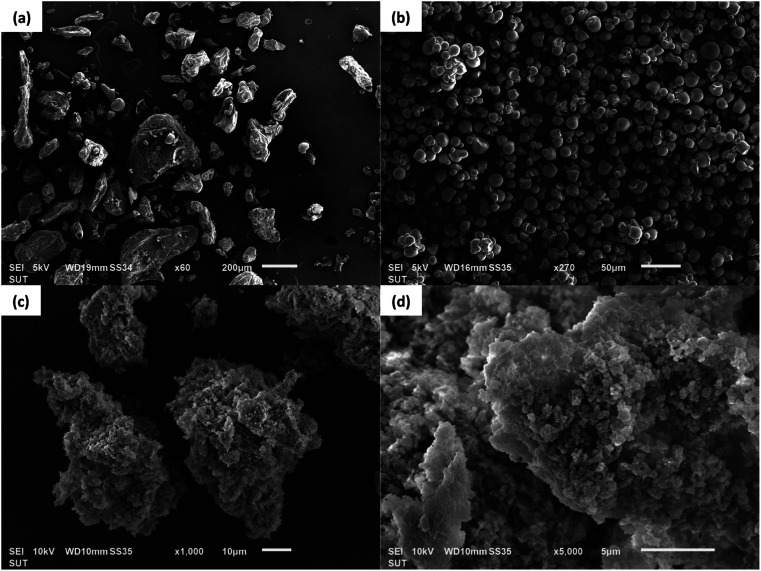
SEM photomicrographs of (a) native papain particles, (b) native ACS particles, (c and d) ACS/papain composites.

The morphological images of the native papain particles, native ACS particles, and ACS/papain composites under a starch concentration of 30 mg mL^−1^, starch volume of 7 mL, and Tween 80 concentration of 3% v/v were obtained using scanning electron microscopy, as depicted in Fig. 7. The native papain particles typically exhibit indistinct shapes (Fig. 7(a)). However, after undergoing precipitation with ethanol, their shape and size transform into spherical and decreased in size from micro to nano levels (Fig. 1). In the case of ACS, the morphological characteristics showed elliptical, kettledrum, or spherical truncated shapes, with some damaged granule particles observed (Fig. 7(b)). Alternatively, the ACS/papain composites appear as rough, indistinct clusters, with their surface featuring small starch nanoparticles clustered together (Fig. 7(c) and (d)), which is similar to the morphology observed in the work by Li *et al.*^21^ who encapsulated zeaxanthin with corn starch using a one-step antisolvent precipitation method.

## 4. Conclusion

The preparation of ACS/papain composites *via* the encapsulation of papain within ACS with a low degree of substitution (DS = 0.037) using the stepwise antisolvent precipitation method was achieved. The study of the effect of starch concentration, starch solution volume, and surfactant type and concentration could determine the suitable conditions to achieve a high encapsulation efficiency and loading capacity, with minimal loss of enzyme activity. The findings revealed that while surfactants facilitate the encapsulation of papain, higher concentrations of surfactant impacted the activity of the enzyme. Tween 80, in particular, achieved the highest encapsulation efficiency, although it led to a notable decrease in the activity of papain. Alternatively, Tween 20 better preserved the activity of papain compared to Tween 80, but its encapsulation efficiency remained lower. The formation of the ACS/papain composites was successfully confirmed by SEM imaging, fluorescence spectroscopy, and FTIR spectroscopy.

These results underscore the preparation and characterization of acetylated starch/papain composites by encapsulating papain within acetylated cassava starch. The results regarding the encapsulation efficiency are promising. However, a limitation of this study is the decrease in papain activity, indicating that the surfactant selection and encapsulation conditions should be further investigated to better preserve the enzyme functionality. The stability, release profile, and biological test studies of the encapsulated papain are recommended for future research.

## Data availability

All data generated or analyzed during this study are included in this published article.

## Author contributions

The conceptualization of the study was conducted by S. B. and L. W., with methodology developed by both authors as well. S. B. carried out the investigation and data curation were done by S. B. and L. W. The original draft was prepared by S. B., and the review and editing were performed by S. B., H. H., and L. W. Visualization was also handled by S. B. The project was supervised and administered by L. W., who also acquired the necessary funding. All authors have read and agreed to the published version of the manuscript.

## Conflicts of interest

There are no conflicts to declare.
